# Assessing the Effectiveness of Tranexamic Acid in Hip Fracture Management: A Systematic Review

**DOI:** 10.7759/cureus.100360

**Published:** 2025-12-29

**Authors:** Mushtaq Ahmed, Mahnoor Qureshi, Mohammad M Khan, Irfan Ullah

**Affiliations:** 1 Trauma and Orthopaedics, Airedale NHS Foundation Trust, Steeton, GBR; 2 Emergency Medicine, Mid Yorkshire Teaching NHS Trust, Wakefield, GBR; 3 Medicine, California Institute of Behavioral Neurosciences & Psychology, Fairfield, USA; 4 Emergency Medicine, Shaheed Mohtarma Benazir Bhutto Institute of Trauma, Karachi, PAK

**Keywords:** blood transfusion need, dosage regimen, patient-centred outcome, tranexemic acid, traumatic hip fracture

## Abstract

Hip fractures often lead to significant blood loss and transfusion requirements in the older population. Tranexamic acid (TXA) is used to control bleeding, but its optimal dose, timing, and broader clinical benefits remain unclear. A systematic search of PubMed, ScienceDirect, the Cochrane Library, Ovid-EMBASE, and EBSCOhost was performed for studies published between 2019 and 2024. Eligible studies included adults with traumatic hip fractures (intra- or extracapsular) treated with TXA. In total, 14 studies met the inclusion criteria, including randomised controlled trials, cohort studies, and meta-analyses. Findings were synthesised narratively due to heterogeneity in interventions and outcomes. TXA consistently reduced intraoperative and postoperative blood loss and lowered transfusion needs, with reductions up to 46% compared with controls. Across various dosing strategies, i.e., preoperative, divided doses, or topical, no significant increase in thromboembolic complications was reported. Limited but favourable evidence suggested improvements in pain, functional recovery, and hospital stay. Benefits appeared more pronounced in extracapsular fractures, where hidden blood loss is typically greater. TXA is a safe and effective adjunct in hip fracture surgery, significantly reducing blood loss and transfusion requirements without increasing the risk of thromboembolism. Standardised dosing protocols are needed, and further research should focus on long-term recovery and tailoring treatment to the type of fracture and patient characteristics.

## Introduction and background

Hip fracture incidents and complications are rising to concerning levels globally. By 2050, the global incidence of hip fractures is projected to reach 6.3 million annually [1]. These injuries are associated with significant morbidity and mortality. Hospital mortality ranges from 4.5% to 6.6%, rising to nearly 25% within one year of fracture [2,3].

Hip fractures are categorised into two main types: extracapsular and intracapsular. This classification is based on the fracture’s location in relation to the hip joint capsule on the femoral neck. Extracapsular fractures include trochanteric fractures, while intracapsular fractures are further divided into two types, namely, fractures of the femoral head and fractures of the neck of the femur.

**Figure 1:**
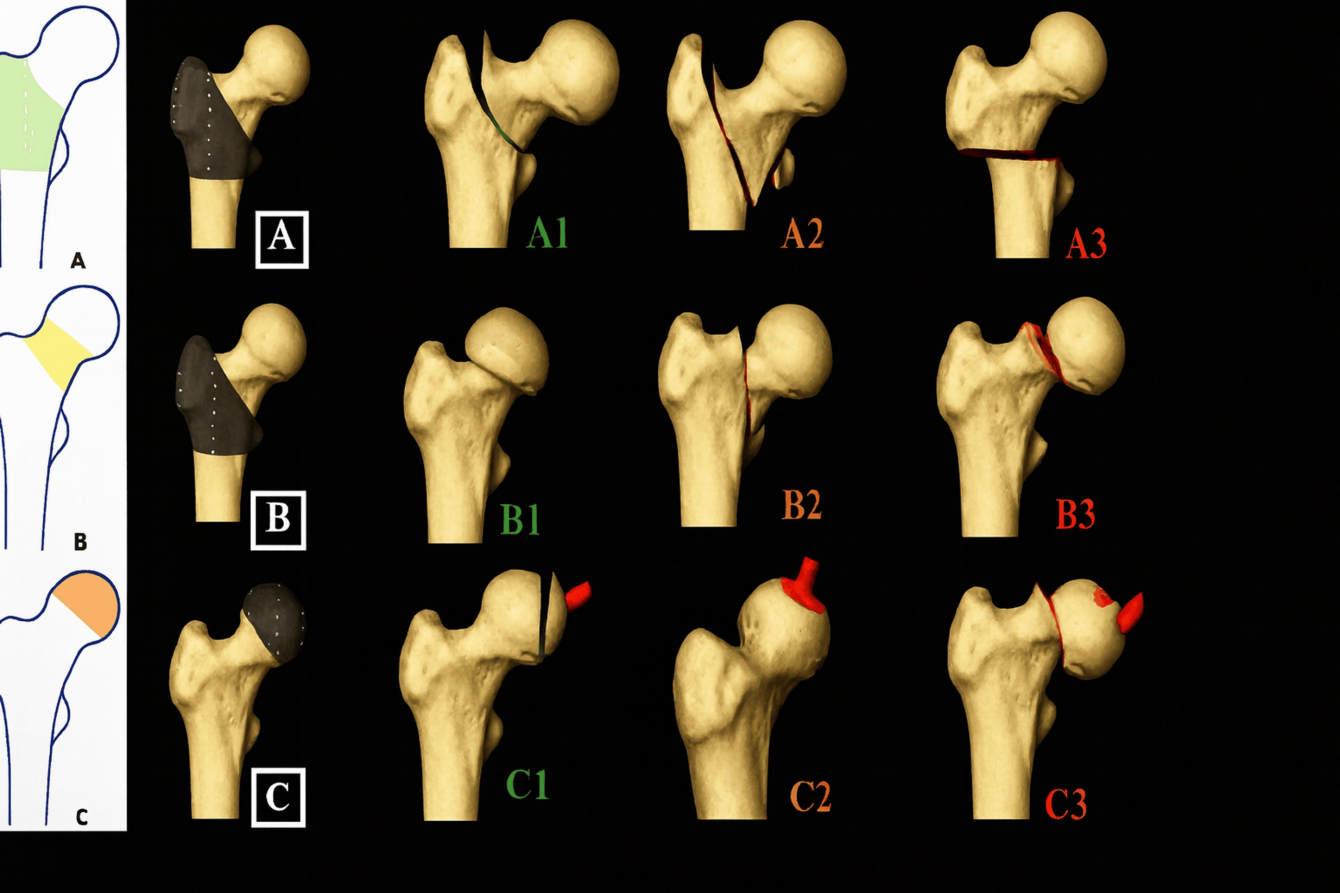
AO classification of proximal femur fractures. Image reproduced from Gallo et al. [4] under the CC BY-NC-ND 4.0 license.

The AO Classification for proximal femur fractures includes the following categories [4], as shown in Figure 1: (1) Type A: simple pertrochanteric A1, multifragmentary pertrochanteric A2, intertrochanteric/reverse oblique A3. (2) Type B: femoral neck subcapital, minimal displacement B1, transcervical B2, and subcapital, displaced B3. (3) Type C: femoral head split C1, femoral head depression C2, and fractures of the femoral head.

Different types of fractures result in varying amounts of blood loss, ranging from 800 mL to 2,200 mL. The average haemoglobin levels drop by 31 g/L in intertrochanteric fractures and 18 g/L in intracapsular fractures [5]. Anaemia is common in this group of people, and it makes recovery take longer, leads to prolonged hospital stays, and causes more deaths. In a previous study, 44% of patients had anaemia upon admission, and 87% developed it after surgery [5].

Allogenic blood transfusion is often considered one of the most effective treatments for anaemia; however, it has significant complications. For example, patients with hip fractures who undergo allogenic blood transfusion have a 35% increased likelihood of developing bacterial infections and a 52% heightened risk of pneumonia, as well as experiencing immune reactions and financial burdens [6,7]. As a result, current guidelines recommend careful transfusion protocols [7,8].

The varying bleeding patterns associated with different fracture types complicate the management of perioperative blood loss. Extracapsular fractures typically involve significant hidden blood loss before surgery, whereas intracapsular fractures usually show a reduction in haemoglobin due to surgical trauma [8]. By lowering fibrinolysis, the synthetic antifibrinolytic drug tranexamic acid (TXA) lowers bleeding during and after surgery [9]. It also prevents plasminogen activation, which helps maintain stable clots. Meta-analyses [9,10] have demonstrated that TXA reduces transfusion requirements by 46% in individuals with hip fractures, without significantly increasing the risk of thromboembolic events.

TXA is crucial for the management of perioperative bleeding following hip surgery and hip fracture [11]. It is highly effective in extracapsular hip fractures, where occult blood loss accounts for approximately 60% of overall blood loss [11]. Additionally, TXA is a reasonably cost-effective treatment that does not significantly increase the risk of venous thromboembolism (VTE) [12,13]. While TXA has demonstrated considerable efficacy, several unresolved challenges hinder its clinical application. Hip fractures can manifest in different forms, leading to uncertainty about optimal dosing strategies and the most effective timing of TXA administration, i.e., before, during, or after surgery. This review aims to address current uncertainties by synthesising evidence across different fracture types and surgical techniques, while highlighting the limited clarity regarding how TXA influences key patient-centred outcomes, including functional recovery, length of hospital stay, quality of life, morbidity, mortality, and transfusion requirements.

Research questions and objectives

This review examines the role of TXA in the surgical management of hip fractures, focusing on its dosage, timing, and overall clinical effectiveness. The primary objective is to investigate the impact of various dosing regimens and routes of administration on perioperative blood loss and transfusion requirements in both intracapsular and extracapsular fractures. Beyond surgical outcomes, the review also considers patient-centred measures, including postoperative pain, mobility, functional recovery, length of hospital stays, morbidity, and mortality. Finally, particular emphasis is placed on evaluating TXA’s safety, particularly its association with thromboembolic risk in elderly populations to clarify its role in evidence-based perioperative care.

## Review

Methodology

Literature Search Strategy

We conducted a comprehensive review of the existing literature on the efficacy of TXA in the management of hip fractures, with an emphasis on the appropriate dosage, timing of administration, transfusion rates, and patient-centred outcomes. The search methodology enabled the identification of all pertinent publications by meticulously selecting appropriate Medical Subject Headings (MeSH) terms and keywords. The primary search terms included “Tranexamic Acid” OR “TXA” AND “Hip Fracture” OR “Neck of Femur Fracture” AND “Optimal Dosage” OR “Dosage” OR “Drug Administration” AND “Transfusion Rate” AND “Blood Loss” AND “Hemostasis” AND “Patient-Centered Outcome” OR “Recovery” AND “Postoperative Pain” AND “Mobility” AND “Morbidity” AND “Mortality.”

We modified these terms to encompass both general and specialised uses of TXA in the treatment of traumatic hip fractures. We employed Boolean operators and MeSH subheadings to enhance the specificity of the outcomes. We examined five principal medical databases, namely, PubMed, ScienceDirect, Cochrane Library, Ovid-EMBASE, and EBSCOhost. We systematically analysed each database in English, and the publication dates spanned 2019 to 2024. This ensured that the information was current and beneficial.

*Inclusion and Exclusion Criteria*

The study focused on individuals aged 19 years and older who suffered traumatic hip fractures. Cohort studies, case series, case reports, randomised controlled trials (RCTs), non-randomised controlled trials, and meta-analyses represent crucial methodologies in research. Both summaries and full-text articles were considered for comprehensive coverage. The included research examined whether TXA could help heal damage caused by hip fractures. Previous studies have explored various aspects of TXA use, including dosing regimens, timing of administration, transfusion rates, intraoperative blood loss, and patient-centred outcomes such as postoperative recovery, pain, morbidity, and mortality. Studies involving TXA in elective hip procedures, animal models, revision or periprosthetic fractures, pathological fractures, or patients with concomitant fractures at other anatomical sites were excluded. Additionally, duplicate articles identified across multiple databases were manually screened and removed to minimise redundancy.

*Study Selection Process: Data Extraction*

After the initial search, two readers reviewed the titles and abstracts of the studies to identify relevant articles. Subsequently, we reviewed and selected studies based on defined eligibility criteria. The goal of data extraction was to look at the characteristics of studies (design, sample size, fracture type), details of the TXA intervention (dose, route, timing), outcomes related to transfusion needs, amount of blood loss, safety endpoints (especially thromboembolic complications), and patient-centred measures (functional recovery, length of hospital stay, morbidity, mortality). Due to the variety of study designs and outcome measures, the results were compiled using a narrative synthesis approach. We supported safety and efficacy outcomes by focusing on quantitative data from RCTs and meta-analyses.

Data Synthesis Method

A well-defined search strategy was employed to synthesise data from the available literature. The goal was to capture all relevant studies while minimising bias. This process involved identifying key concepts and utilising specific search terms related to the central themes of this review. The main components of the search strategy are presented below.

Tranexamic acid: Tranexamic acid is a key focus of this review, given its role in managing blood loss, particularly in patients with hip fractures. The search terms for TXA included (“tranexamic acid”[MeSH Terms] OR Tranexamic acid [Text Word] OR TXA [All Fields]). This comprehensive search was designed to capture all available studies and data on TXA, ensuring that no relevant research was overlooked.

Hip fracture: Hip fractures are a critical area of focus, especially in relation to the use of TXA. The search for studies on hip fractures included several specific sub-topics to refine the results and capture relevant studies across different aspects of hip fracture management. The search terms used were: (“Tranexamic Acid”[MeSH]) AND (“Hip Fractures/blood”[MeSH] OR “Hip Fractures/complications”[MeSH] OR “Hip Fractures/drug therapy”[MeSH] OR “Hip Fractures/economics”[MeSH] OR “Hip Fractures/epidemiology”[MeSH] OR “Hip Fractures/etiology”[MeSH] OR “Hip Fractures/genetics”[MeSH] OR “Hip Fractures/history”[MeSH] OR “Hip Fractures/metabolism”[MeSH] OR “Hip Fractures/mortality”[MeSH] OR “Hip Fractures/nursing”[MeSH] OR “Hip Fractures/physiopathology”[MeSH] OR “Hip Fractures/psychology”[MeSH] OR “Hip Fractures/rehabilitation”[MeSH] OR “Hip Fractures/surgery”[MeSH] OR “Hip Fractures/therapy”[MeSH]). These terms were chosen to cover all relevant studies, from the basic mechanisms of hip fractures to the impact of treatments and recovery outcomes.

Blood transfusion, blood loss, and haemostasis: The following search terms were used: (“Blood Transfusion”[MeSH]) AND (“Blood Loss, Surgical”[MeSH] OR “Hemostasis, Surgical”[MeSH] OR “Blood Transfusion, Autologous”[MeSH] OR “Postoperative Haemorrhage”[MeSH]). These terms enabled the inclusion of studies on the broader context of surgical interventions and haemostasis, providing a comprehensive view of how TXA affects bleeding control during surgery.

Drug dosage and optimal dosage: Another key aspect of the review was to identify studies discussing the optimal dosages of TXA and its administration. It is essential to determine the optimal dosage to achieve optimal patient outcomes. The following search terms were used to target relevant studies: “Drug Evaluation”[MeSH] AND “Drug Administration Routes”[MeSH] AND “Administration, Cutaneous”[MeSH]. These terms were carefully selected to identify studies assessing drug dosage and routes of administration that optimise the effects of TXA, with a focus on both efficacy and safety.

Patient-centred outcomes, mortality, morbidity, mobility, and postoperative pain: The ultimate goal of any medical intervention is to improve patient outcomes, including mortality, morbidity, mobility, and postoperative pain. A robust search strategy was employed to capture studies that focused on these outcomes in patients receiving TXA for hip fractures. The terms used were: ((((“Patient Outcome Assessment”[MeSH]) AND “Mortality”[MeSH]) AND “Morbidity”[MeSH]) AND (“Pain, Postoperative/blood”[MeSH] OR “Pain, Postoperative/classification”[MeSH] OR “Pain, Postoperative/complications”[MeSH] OR “Pain, Postoperative/diet therapy”[MeSH] OR “Pain, Postoperative/drug therapy”[MeSH] OR “Pain, Postoperative/economics”[MeSH] OR “Pain, Postoperative/epidemiology”[MeSH] OR “Pain, Postoperative/etiology”[MeSH] OR “Pain, Postoperative/history”[MeSH] OR “Pain, Postoperative/microbiology”[MeSH] OR “Pain, Postoperative/mortality”[MeSH] OR “Pain, Postoperative/pathology”[MeSH] OR “Pain, Postoperative/physiopathology”[MeSH] OR “Pain, Postoperative/rehabilitation”[MeSH] OR “Pain, Postoperative/surgery”[MeSH] OR “Pain, Postoperative/therapy”[MeSH])) AND “Range of Motion, Articular”[MeSH]. This term structure was designed to cover a wide range of patient-centred outcomes, from pain management to long-term functional mobility, providing a comprehensive overview of the treatment effectiveness.

Database-Specific Search Details

PubMed: Searched for combination terms regarding TXA and hip fractures, applying filters for the English language, publication years from 2019 to 2024, and adult patients aged 19 years and older. Multiple RCTs, meta-analyses, and observational studies were searched.

ScienceDirect: Filtered by publication period from 2019 to 2024, utilised keyword combinations “TXA acid AND hip fracture AND optimal dosage AND blood loss OR transfusion rate AND patient-centred outcomes.”

Cochrane Review: Utilised keyword logic to identify targeted clinical trials and systematic reviews, subsequently locating one Cochrane review and 17 clinical trials on the topic.

Ovid-EMBASE: Restricted to the English language and current publications; encompassed all domains focusing on TXA, hip fractures, dosage, blood loss, haemostasis, and patient outcomes.

EBSCOhost: Restricted to full-text articles from the last five years; comprehensive search on TXA in hip fractures or femoral neck injuries.

Study Selection and Quality Appraisal

Using specific criteria developed for systematic reviews, we assessed the methodological quality and relevance of relevant papers identified through database searches. When appropriate, quality standards for observational studies and risk-of-bias criteria for randomised trials were used to assess the reliability of the synthesis.

Data Extraction

Two reviewers used a standard form that had already been set up to get their own data. Study design, sample size, type of fracture, TXA dosage and timing, transfusion-related outcomes, adverse events, and patient-centred metrics were critical pieces of information. If reviewers disagreed, they either addressed the issue themselves or consulted a third reviewer. This stringent procedure ensured reliability and transparency of the synthesis process.

Results

**Figure 2:**
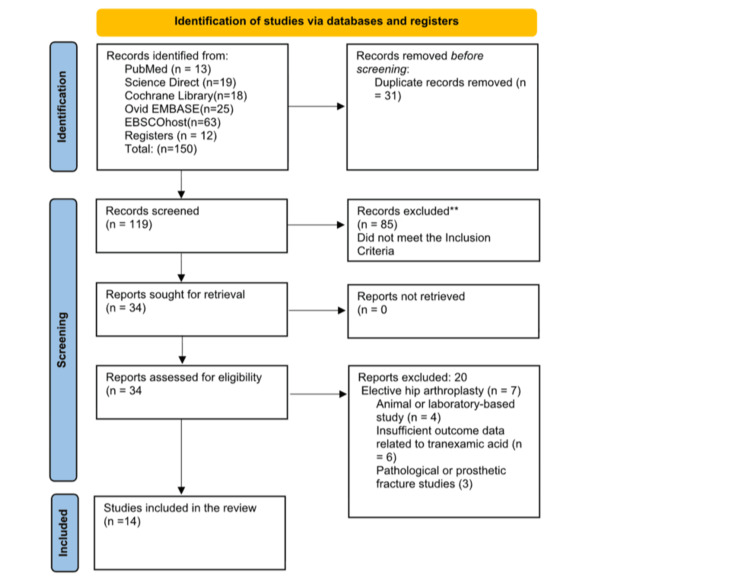
Preferred Reporting Items for Systematic Reviews and Meta-Analyses (PRISMA) flowchart illustrating the selection process of studies.

We identified 150 records through database searches and other sources. We found 138 records from five electronic databases (PubMed, ScienceDirect, the Cochrane Library, Ovid-EMBASE, and EBSCOhost). We also identified 12 additional records through manual review of reference lists. After removing 31 duplicate records, 119 unique records remained. We reviewed the titles and abstracts of these records. We excluded 85 because they did not meet the inclusion criteria (e.g., studies of unrelated therapies, elective operations, or outcomes that were not relevant). We reviewed 34 full-text articles to determine eligibility. Of these, 20 were not included for the following reasons: elective hip arthroplasty or revision surgeries (n = 7), studies conducted on animals or in laboratories (n = 4), insufficient outcome data concerning TXA (n = 6), and studies concerning pathological or periprosthetic fractures (n = 3). Ultimately, 14 studies met all the requirements and were included in the qualitative synthesis. These studies were also included in the quantitative synthesis (meta-analysis) when sufficient outcome data were available (Figure 2).

Characteristics of Included Studies

A total of 14 primary studies were included. Collectively, these studies encompassed over 1,500 elderly patients undergoing surgical intervention for either intracapsular or extracapsular hip fractures. The broad representation of both fracture types enables a more nuanced understanding of TXA’s effectiveness across various surgical contexts [14].

Tranexamic Acid Dosage Regimens and Timing of Administration

One consistent finding across the reviewed studies was variation in the timing and dose of TXA administration. Single-dose protocols, typically involving a 1 g intravenous (IV) dose administered preoperatively, significantly reduced intraoperative and postoperative blood loss and transfusion rates, without an increase in thromboembolic events [15]. Regimens using two doses of 15 mg/kg IV, administered before surgery and repeated three hours postoperatively, showed a significant decrease in postoperative haemoglobin drop and transfusion requirements [16]. Comparisons of a single 2 g bolus versus two 1 g doses (one pre-incision and one post-incision) indicated that the split-dose approach offered improved intraoperative blood conservation and overall haemostatic efficacy [17]. In a double-masked RCT, topical administration of TXA was found to be equally effective as IV delivery in reducing transfusion needs, with fewer systemic side effects, suggesting a safer option for patients at higher risk [18]. Table 1 shows the clinical studies done on the dosage and timing of TXA in hip fractures.

**Table 1:** Clinical studies on TXA timing and dosage on hip fracture management. TXA: tranexemic acid; RCT: randomised controlled trial; IV: intravenous

Study	Design	Sample size	Fracture type	TXA regimen	Timing	Outcome
Luo et al., 2023 [18]	RCT	98	Intertrochanteric	1 g IV preoperatively	Preoperative	Reduced blood loss
Stojadinovic et al., 2022 [19]	RCT	90	Hip fracture	1 g IV preoperatively	Preoperative	Reduced blood loss and thromboembolism
Narkbunnam et al., 2021 [20]	RCT	120	Femoral neck	1 g IV + 1 g postoperatively	Preoperative and postoperative	Lower transfusion rate
Khatib et al., 2024 [14]	RCT	80	Intracapsular	Single 1 g IV preoperatively	Preoperative	Reduced transfusion
Ashkenazi et al., 2020 [3]	RCT	110	Hip hemiarthroplasty	IV before incision	Intraoperative	Effective
Yee et al., 2022 [21]	RCT	85	Intertrochanteric	Topical	Topical	Effective topical route
Van Rijckevorsel et al., 2022 [22]	Cohort	112	Hip hemiarthroplasty	1 g IV	Preoperative	Reduced transfusion
Ekinci et al., 2022 [7]	RCT	75	Intertrochanteric	1 g IV preoperatively	Preoperative	Reduced total blood loss
Gausden et al., 2016 [8]	RCT	150	Mixed	1 g IV	Preoperative	Protocol trial
Zufferey et al., 2010 [23]	RCT	87	Hip fracture	1 g IV	Preoperative	Reduced transfusion
Shichman et al., 2021 [24]	Cohort	91	Total hip arthroplasty	1 g IV	Preoperative	Reduced transfusion
Huynh et al., 2021 [11]	Cohort	105	Hip fracture	1 g IV	Preoperative	Reduced blood loss
Cheung et al., 2020 [6]	Observational	4800	Hip fracture	1 g IV	Preoperative	Safe and effective
Moran et al., 2022 [25]	Cohort	198	Geriatric hip fracture	1 g IV at admission	Admission	Lower transfusion rate

Impact of Tranexamic Acid on Blood Loss and Transfusion Requirements

Several studies have demonstrated that administering TXA is associated with a significant reduction in total blood loss and transfusion requirements. For instance, across eight RCTs, the need for blood transfusions decreased by 18% [18]. Moreover, the average transfusion reduction rate was 41% in systematic reviews and meta-analyses of major studies [15,26,27]. On average, total blood loss was reduced by approximately 350 mL, with the effect being particularly pronounced in extracapsular fractures, which are associated with greater intraoperative bleeding [14]. These findings have important clinical implications for older patients, who are more susceptible to complications, prolonged hospital stays, and delayed recovery following significant blood loss or transfusion. The observed reductions in blood loss and transfusion rates suggest that TXA plays a crucial role in improving perioperative outcomes in this vulnerable population.

Safety Outcomes: Thromboembolic and Other Adverse Events

Several high-quality studies have examined the thromboembolic safety of TXA, a key concern in its perioperative use. A meta-analysis found no significant differences in the incidence of deep vein thrombosis, pulmonary embolism, or cardiovascular events between TXA-treated and control groups [25]. Likewise, results from an extensive multicentre cohort study involving over 500 elderly patients with hip fractures showed that TXA can be safely administered to frail patients with comorbidities, provided careful perioperative monitoring is performed [20].

Patient-Centred Outcomes

Although often underreported, some studies evaluated functional recovery and quality of life after surgery. TXA use was associated with better haemoglobin preservation, which helped reduce hospital stays and enabled earlier postoperative mobilisation [28]. However, the review emphasised the need for more RCTs that include standardised patient-centred outcomes, such as pain scores, mobility assessments, quality of life measures (e.g., EuroQol 5 Dimensions), and time to regain independence.

Subgroup Analyses: Intracapsular Versus Extracapsular Fractures

The reviewed literature supports the hypothesis that the effectiveness of TXA varies by fracture type. Extracapsular fractures, including intertrochanteric and subtrochanteric fractures, are associated with greater soft tissue damage and hidden blood loss, and TXA administration in these cases consistently reduced transfusion requirements and overall haemorrhage. Improvements were also observed in intracapsular fractures, although to a greater extent, likely due to the surgical approach and limited haematoma formation. These findings emphasise the importance of tailoring TXA regimens to the type of fracture and expected blood loss [24].

Systematic Reviews on Tranexamic Acid Use in the Management of Hip Fractures

**Figure 3:**
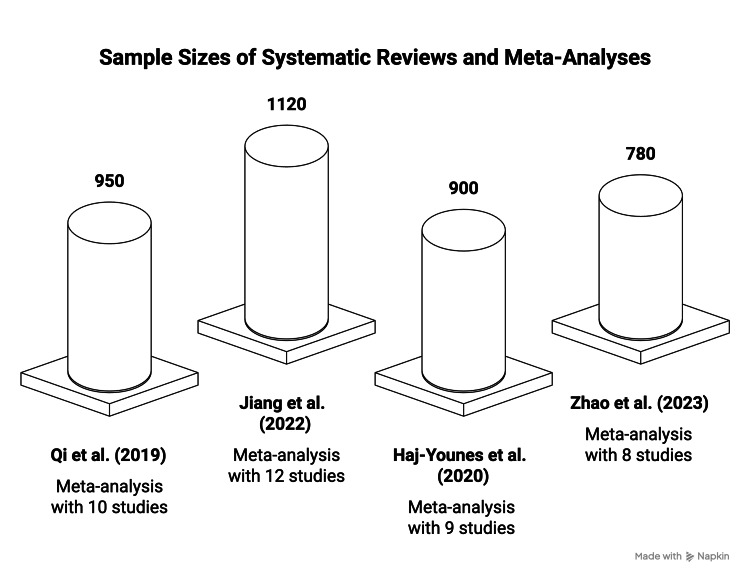
Sample size and number of systemic reviews on TXA use in the surgical management of hip fractures. Qi et al. (2019) [28]; Jiang et al. (2022) [12]; Haj-Younes et al. (2020) [10]; Zhao et al. (2023) [28]. TXA: tranexemic acid

Figure 3 displays four key systematic reviews and meta-analyses that investigated the use of TXA in the surgical treatment of hip fractures. The vertical bar represents the sample size of included studies. The study [12] consists of a large sample of 1,120 patients and is among the most comprehensive, demonstrating its extensive scope. The graph compares the scale and depth of evidence from different studies on the safety and effectiveness of TXA in the surgical management of hip fractures. Table 2 shows the characteristics of the same systematic reviews and meta-analyses.

**Table 2:** Systematic reviews on TXA use in hip fractures. TXA: tranexemic acid; IV: intravenous; VTE: venous thromboembolism; DVT: deep vein thrombosis; PE: pulmonary embolism

Study	Type	Included studies	Sample size	Focus	Outcomes
Qi et al. (2019) [28]	Systematic review and meta-analysis	10	950	Efficacy and safety of IV TXA in hip fracture surgery	Reduced transfusion; no VTE increase
Jiang et al. (2022) [12]	Systematic review and meta-analysis	12	1120	TXA in intertrochanteric fractures with intramuscular fixation	Significant reduction in blood loss
Haj-Younes et al. (2020) [10]	Systematic review and meta-analysis	9	900	TXA effectiveness and thromboembolic risks	TXA effective; no increased DVT/PE
Zhao et al. (2023) [27]	Systematic review and meta-analysis	8	780	TXA safety in the elderly with femoral neck fracture and arthroplasty	Safe with reduced transfusions

Risk of Bias Assessment of Included Studies

Table 3 outlines the risk of bias assessment according to the Cochrane Risk of Bias tool, which assesses selection, performance, detection, attrition, reporting, and other sources of bias. A domain-based approach was adapted for cohort studies and systematic reviews.

**Table 3:** Risk of bias assessment of included studies. RCT: randomised controlled trial

Study	Study type	Selection bias	Performance bias	Detection bias	Attrition bias	Reporting bias	Other bias
Luo et al., 2020 [26]	Systematic review	Low	Low	Low	Low	Low	High (heterogeneity across included studies)
Miangul et al., 2023 [13]	Meta-analysis	Low	Low	Low	Low	Low	High (publication bias possible)
Kim et al., 2022 [15]	Cohort study	Moderate	Low	Low	Moderate	Low	High (non-random selection)
Blumenthal et al., 2024 [5]	Cohort study	Moderate	Low	Low	Moderate	Low	High (non-random selection)
Qi et al., 2019 [28]	Systematic review	Low	Low	Low	Low	Low	High (heterogeneity across included studies)
Jiang et al., 2022 [1]	Meta-analysis	Low	Low	Low	Low	Low	High (publication bias possible)
Haj-Younes et al., 2020 [10]	Meta-analysis	Low	Low	Low	Low	Low	High (publication bias possible)
Zhao et al., 2023 [27]	Systematic review	Low	Low	Low	Low	Low	High (heterogeneity across included studies)
Cheung et al., 2020 [6]	Cohort study	Low	Low	Low	Low	Low	High (non-random selection)
Stojadinovic et al., 2022 [19]	RCT	Low	Low	Low	Low	Low	None
Xie et al., 2024 [29]	RCT	Low	Low	Low	Low	Low	None
Khatib et al., 2024 [14]	RCT	Low	Low	Low	Low	Low	None
Yee et al., 2022 [21]	RCT	Low	Low	Low	Low	Low	None
Ashkenazi et al., 2020 [2]	RCT	Low	Low	Low	Low	Low	None

Perspectives of Two Reviewers in Academic Contexts

Reviewer 1 indicates that the data extraction process is comprehensive and reliable. This clarifies the research methodology. Employing standardised procedures and resolving disagreements through discussion or arbitration improves the reliability of the results. The study comprises 14 original research articles that examine the effects of TXA on hip fracture surgery across various dose regimens, timing, and fracture types. Reviewer 1 emphasises the importance of prioritising safety outcomes and advocates further research, particularly on patient-related complications. Conversely, Reviewer 2 is likely to focus on the differences in how TXA was administered across the study. Some regimens have been effective but need to be adjusted to suit the specific surgical circumstances.

It is clinically essential to examine the differences in outcomes, especially given that TXA is more effective in extracapsular fractures, to identify ways to enhance its efficacy. If TXA addressed thromboembolic safety and emphasised that there were no adverse events, particularly in patients at risk, Reviewer 2 would feel more confident that it is safe. This indicates that TXA can be safely administered to older individuals with other health conditions. Both authors agree that more RCTs focusing on patient-centred outcomes are needed to properly assess the effectiveness of TXA in treating hip fractures.

Discussion

Consistent outcomes across various trial designs indicate that TXA is a significantly beneficial adjunct in the management of haemorrhage following hip fractures. TXA has been shown to reduce the need for allogenic transfusions and indirectly facilitates healing and may reduce complications. A meta-analysis [26] provides strong evidence of TXA’s effectiveness, particularly in extracapsular fractures. These findings are crucial for improving surgical safety, particularly for older individuals, who are more vulnerable to the adverse effects of anaemia and blood transfusions.

There is still no consensus on the optimal TXA treatment for individuals with hip fractures. Although split-dose and dual-phase administration (preoperative and postoperative ) have demonstrated greater efficacy, single-dose protocols are straightforward to implement and still offer benefits. Topical application of TXA may be preferable for patients at high risk of systemic complications or for whom intravenous antifibrinolytics are contraindicated. More head-to-head trials are needed to identify the most cost-effective and clinically effective protocols for various patient groups. This review is significant in demonstrating that TXA is safe for elderly patients with hip fractures, including those at risk of thromboembolic events. Although clinicians should continue to evaluate individual patient risk profiles, current evidence does not support withholding TXA solely due to concerns about VTE in routine care. Despite evidence of TXA’s physiological benefits, the mechanisms by which it affects function and quality of life remain unclear. Future studies should incorporate standardised instruments for assessing mobility, postoperative pain, cognitive recovery, and long-term independence, as these metrics are of utmost importance to patients and caregivers.

Limitations

Despite the comprehensive nature of this review, several limitations must be acknowledged. The inclusion of studies with varying research designs, such as cohort studies, meta-analyses, and RCTs, introduced methodological heterogeneity in patient selection, outcome assessment, and data interpretation. This variation necessitated a narrative synthesis rather than a complete meta-analysis. Furthermore, there was significant inconsistency in TXA dosing regimens and administration protocols across studies, with differences in route (IV versus topical), dosage (fixed versus weight-based), and timing (preoperative, perioperative, or postoperative) [21,30,31]. Such variability likely contributed to discrepancies in reported outcomes. Another limitation lies in the limited focus on patient-centred outcomes. Although some studies assessed parameters such as functional recovery, hospital stay, or postoperative mobility, these were often secondary outcomes and inconsistently defined, making it challenging to draw conclusions about the broader impact of TXA on quality of life [29]. In addition, subgroup analyses exploring variations by age, sex, comorbidities, or fracture type (intracapsular versus extracapsular) were scarce, thereby limiting the generalizability of the findings to diverse patient populations [30]. Publication bias may also have influenced the results, as studies reporting positive effects are more likely to be published and thus more likely to be cited. Unpublished or grey literature was not included, despite extensive database searches. Lastly, most studies have focused on short-term perioperative outcomes, such as transfusion rates and changes in haemoglobin, while overlooking longer-term endpoints, including 30-day morbidity, functional independence, and survival, which are particularly relevant in elderly hip fracture patients [29]. This short follow-up duration limits the understanding of TXA’s long-term clinical implications and overall recovery trajectory.

## Conclusions

TXA is a safe and effective intervention to reduce perioperative blood loss and transfusion requirements in patients undergoing hip fracture surgery. Both single-dose and multiple-dose regimens have been demonstrated to be effective in various surgical contexts. Significantly, even among older adults with multiple comorbidities, TXA does not seem to substantially raise the risk of thromboembolic complications. These findings support the incorporation of TXA into routine perioperative management for hip fractures, contributing to improved haemoglobin preservation, suggesting potential benefits in patient outcomes during the perioperative period. Nevertheless, the considerable variability in study protocols, patient characteristics, and administration methods highlights ongoing uncertainty regarding the optimal dosage and timing of TXA. Further research is required to determine its effects on broader patient-centred outcomes, including quality of life, pain, mobility, and postoperative recovery. Future studies should focus on standardised trials that directly compare dosing strategies and tailor regimens based on fracture classification (intracapsular versus extracapsular). Additionally, evaluations of cost-effectiveness in resource-limited healthcare settings and investigations into long-term functional recovery are essential. Given the high prevalence of hip fractures worldwide, especially among the ageing population, this research is particularly important. Finally, when incorporated into patient-centred, evidence-based perioperative protocols, TXA is a low-risk, low-cost intervention with high clinical value in hip fracture management.
